# A Comparative Study of Hummingbirds and Chickens Provides Mechanistic Insight on the Histidine Containing Dipeptide Role in Skeletal Muscle Metabolism

**DOI:** 10.1038/s41598-018-32636-3

**Published:** 2018-10-03

**Authors:** E. Dolan, B. Saunders, W. S. Dantas, I. H. Murai, H. Roschel, G. G. Artioli, R. Harris, J. E. P. W. Bicudo, C. Sale, B. Gualano

**Affiliations:** 1 Applied Physiology and Nutrition Research Group, Rheumatology Division; Faculdade de Medicina FMUSP, Universidade de Sao Paulo, Sao Paulo, SP, BR, University of São Paulo, São Paulo, SP Brazil; 2 Junipa Ltd; Newmarket, Suffolk, United Kingdom; 3 School of Biological Sciences, University of Wollongong, Wollongong, Australia; 4 Sport, Health and Performance Enhancement Research Centre; Musculoskeletal Physiology Research Group; School of Science and Technology, Nottingham Trent University, Nottingham, United Kingdom

## Abstract

Histidine containing dipeptides (HCDs) have numerous ergogenic and therapeutic properties, but their primary role in skeletal muscle remains unclear. Potential functions include pH regulation, protection against reactive oxygen/nitrogen species, or Ca^2+^ regulation. In recognition of the challenge of isolating physiological processes *in-vivo*, we employed a comparative physiology approach to investigate the primary mechanism of HCD action in skeletal muscle. We selected two avian species (*i*.*e*., hummingbirds and chickens), who represented the extremes of the physiological processes in which HCDs are likely to function. Our findings indicate that HCDs are non-essential to the development of highly oxidative and contractile muscle, given their very low content in hummingbird skeletal tissue. In contrast, their abundance in the glycolytic chicken muscle, indicate that they are important in anaerobic bioenergetics as pH regulators. This evidence provides new insights on the HCD role in skeletal muscle, which could inform widespread interventions, from health to elite performance.

## Introduction

The histidine containing dipeptide (HCD) carnosine, and its related methylated analogues (anserine and balenine), are formed by β-alanine and L-histidine, and are purported to have pleiotropic ergogenic and therapeutic effects. These include the enhancement of high-intensity exercise performance^1^, protection against the effects of senescence^2^, neuro-protection^3,4^ and tumour inhibition^5^. These influences may occur due to a number of mechanisms, namely intracellular proton buffering, protection against reactive species and/or the regulation of Ca^2+^ transients and sensitivity^6,7^. Their primary role, however, is unknown. This renders targeted intervention difficult, meaning that the full therapeutic and ergogenic potential of these dipeptides remains unexploited. The abundance of HCDs (carnosine) in human skeletal muscle (approximately 20–30 mmol.kgDM^−1^ in *m*. *vastus lateralis*^8^), along with evidence of a positive influence on exercise performance^1^, implies an important role in skeletal muscle metabolism. Elucidation of their primary role in skeletal muscle is challenging, however, as all potential processes to which they may contribute up-regulate concurrently and cooperatively in response to high-intensity activity. Similarly, many of the conditions in which HCDs have been reported to convey a therapeutic benefit are multi-factorial, therefore potentially benefitting from most, or all, of the HCDs purported biological functions.

**Figure 1:**
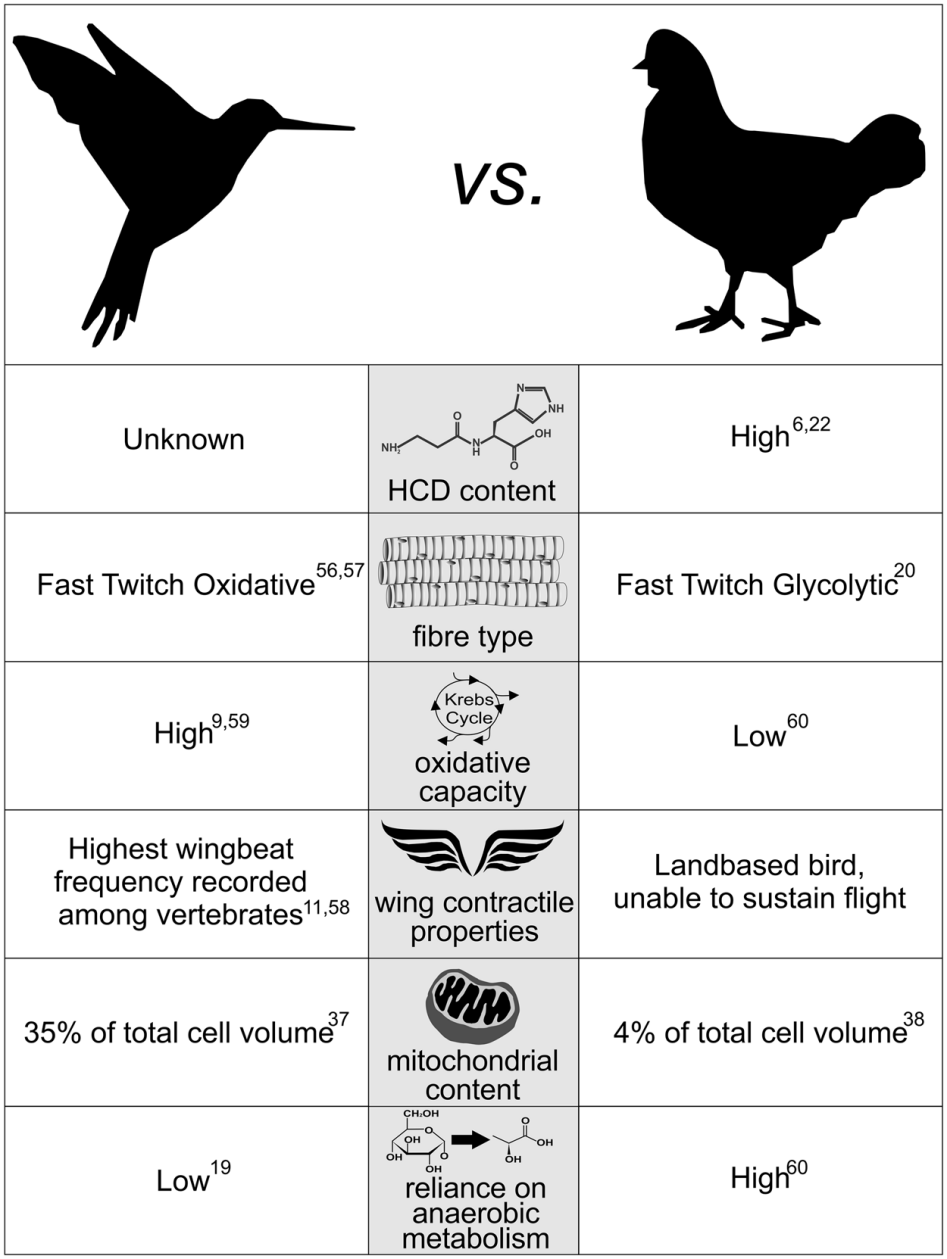
Characteristics of the Hummingbird & Chicken *M*. *Pectoralis*^6,9,11,19,20,22,37,38,56–60^.

In recognition of the challenge of isolating biological processes *in-vivo*, we employed a comparative physiology approach to provide new insight into the primary mechanism of HCD action in skeletal muscle metabolism. More specifically, we selected two physiologically distinct avian species (namely hummingbirds and chickens), whose unique skeletal muscle types represented the extremes of biological processes in which HCDs are most likely to exert their primary influence. An overview of the contrasting characteristics of the hummingbird and chicken *m*. *pectoralis* are described in Fig. 1. Of particular relevance to this investigation, is the hummingbird’s remarkable skeletal muscle characteristics, which gives rise to its unique locomotive ability. Hummingbirds have an outstanding capacity to accelerate and to alter flight speed, trajectory and body orientation^9,10^. They are also capable of beating their wings with sufficient frequency to hover. This characteristic is unique among avian species, and occurs due to their extraordinarily high wing-beat frequency, which is the fastest of all vertebrates^11,12^. This is achieved through highly developed contractile properties, and requires optimised regulation of Ca^2+^ transients and sensitivity^13^. Hummingbirds also have the highest mass-specific metabolic rate of all vertebrates^14^, achieved through an outstanding capacity to deliver, uptake and utilize oxygen^9^, allowing a constant supply of aerobically generated energy to the working muscles^15,16^. This extremely advanced oxidative system, must be accompanied by an equally well-developed system to neutralise the metabolic by-products of oxidative phosphorylation, namely reactive species^17^. Consequently, if the main role of HCDs in skeletal muscle metabolism is to act either in the primary protection against reactive species or in the regulation of calcium transients and sensitivity, then they would be abundantly expressed in the hummingbird flight musculature. In contrast to their outstanding oxidative and contractile properties, hummingbirds have limited capacity for anaerobic metabolism^18,19^, most likely because their aerobic capacity renders anaerobic bioenergetics largely unnecessary. Indeed the phosphofructokinase:lactate dehydrogenase enzyme activity ratio in hummingbirds is far higher than in other vertebrates^19^, indicating that the glycolytic conversion of glucose to pyruvate is designed for complete oxidation through the krebs cycle and electron transport chain, and not for conversion of pyruvate to lactate, as occurs when insufficient oxygen is available for complete oxidative metabolism in the mitochondria. Given that intramuscular acidosis occurs as a result of hydrogen ion accumulation during anaerobic metabolism, the highly advanced aerobic capacities of the hummingbird, and thus, reduced reliance on anaerobic metabolism mean that their flight musculature is not routinely exposed to high acid loads, and therefore has limited requirement for a highly adapted intracellular physicochemical buffering system. Therefore if the primary role of carnosine is to act as an intracellular buffer, high levels would not be required in the highly aerobic hummingbird tissue. The HCD content of the hummingbird flight muscle is, however, unknown.

Conversely, the chicken flight musculature has evolved as a largely vestigial tissue, and is primarily composed of glycolytic muscle fibres^20^, rendering these muscles capable of short bursts of powerful contraction when required (*e*.*g*., to escape imminent danger). They have limited capacity for oxidative metabolism, or muscle contraction and, therefore, they cannot undertake sustained flight. Accordingly, chicken flight is largely dependent on anaerobic energy metabolism. Chickens have previously been reported to have high skeletal muscle HCD content^6,21,22^, and this has been interpreted as implying a primary role for HCDs as intracellular proton buffers^23^. The legs are the primary locomotive unit of this landbased bird, and are known to have a greater oxidative capacity, but lower HCD content than the predominantly glycolytic *m*. *pectoralis*^22^. As such, the chicken *m*. *vastus lateralis* would represent an “intermediate” muscle type between the highly oxidative hummingbird, and highly glycolytic chicken *m*. *pectoralis*.

The primary aim of this study was to measure the HCD content of the flight muscle (*m*. *pectoralis*) in both species, along with the chicken *m*. *vastus lateralis*. Cytochrome C oxidase, subunit IV (COX IV) and lactate dehydrogenase (LDH) content, superoxide dismutase (SOD) activity and *in vitro* muscle buffering capacity (βm), were also measured, and used to provide an indication of the aerobic and anaerobic capacities of the muscle types under investigation, thus offering further mechanistic insight into the role of HCDs in skeletal muscle metabolism.

## Results

### HCD content

**Figure 2:**
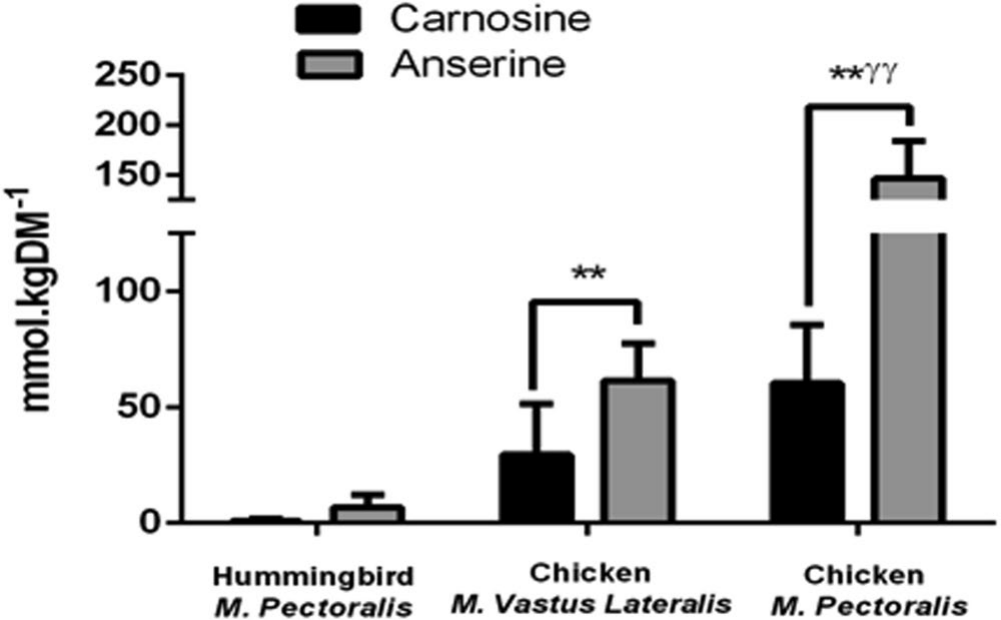
Histidine Containing Dipeptide (HCD) Content of Hummingbird and Chicken. ^**^p < 0.01 from hummingbird *m*. *pectoralis*; ^ƴƴ^ p < 0.01 from chicken *m*. *vastus lateralis*. Hummingbird *m*. *pectoralis* n = 5; chicken *m*. *vastus lateralis* n = 6; chicken *m*. *pectoralis* n = 6.

Total HCD content (*i*.*e*., carnosine + anserine) was significantly different between species (p < 0.001 for all between-muscle sample comparisons; Fig. 2), with the HCD content of hummingbird *m*. *pectoralis* (7.46 ± 2.6 mmol.kgDM^−1^) being substantially lower than both the chicken *m*. *vastus lateralis* (91.18 ± 9.10 mmol.kgDM^−1^) and *m*. *pectoralis* (206.69 ± 17.76 mmol.kgDM^−1^). Consistently higher levels of anserine compared to carnosine were recorded in all tissues (p < 0.001 for all within sample comparisons).

### Muscle characterisation

**Figure 3:**
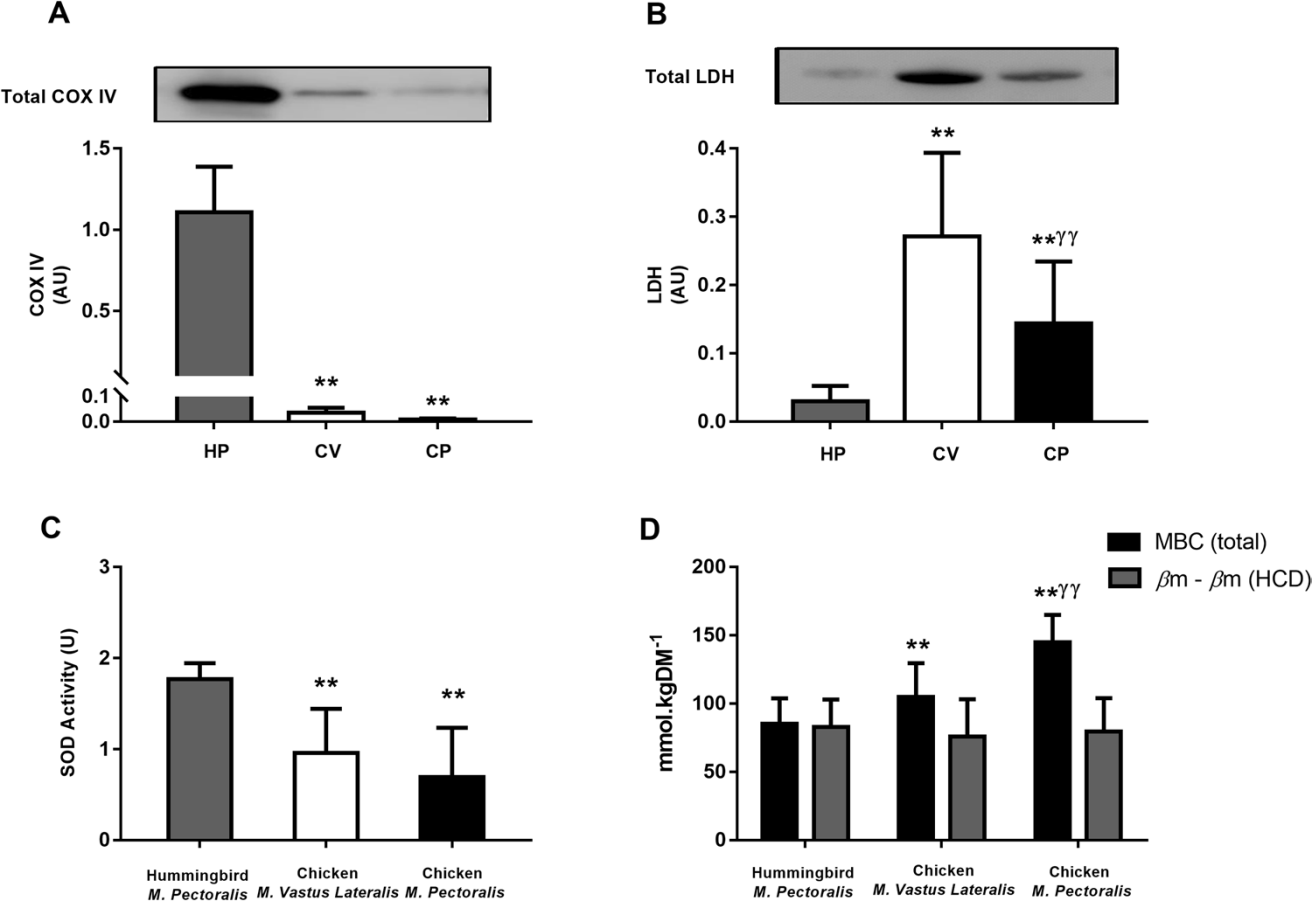
Characteristics of Hummingbird *M*. *Pectoralis* (HP), Chicken *M*. *Vastus Lateralis* (CV) and Chicken *M*. *Pectoralis* (CP). Panel (A) COX IV content; Panel (B) LDH content; Panel (C) Superoxide Dismutase (SOD) Activity; Panel (D) Muscle buffering capacity (βm) represented with and without HCD contribution. **p < 0.01 from hummingbird *m*. *pectoralis*; ^ƴƴ^ p < 0.01 from chicken *m*. *vastus lateralis*; Hummingbird *m*. *pectoralis* n = 5; chicken *m*. *vastus lateralis* n = 6; chicken *m*. *pectoralis* n = 6. The bands shown in panels A and B were loaded in the order described and were cropped from the same location and gel. Full-length blots are presented in Supplementary File 1.

Data on COX IV and LDH content, SOD activity and βm are presented in Fig. 3. Hummingbird *m*. *pectoralis* had substantially higher COX IV content and SOD activity than either of the two chicken muscle samples (p < 0.001 for all between-species comparisons). The difference in COX IV and SOD activity between chicken *m*. *vastus lateralis* and *m*. *pectoralis* were non-significant (p = 0.949 and 0.058). Hummingbird *m*. *pectoralis* had substantially lower LDH content than either chicken muscle type (p < 0.01 for all between species comparisons), while chicken *m*. *vastus lateralis* had more LDH than *m*. *pectoralis* (p < 0.01). βm of the chicken *m*. *pectoralis* was higher than *m*. *vastus lateralis* (p < 0.001), while both chicken samples had a higher βm than the hummingbird *m*. *pectoralis* (both p < 0.01; Fig. 3, Panel D). Calculation of the nonHCD buffering capacity using the Henderson-Hasselbalch equation indicated that the HCD buffering contribution (βm_HCD_) was responsible for all of the reported variation in βm between the three muscle samples (βm_nonHCD_ = 79.2 ± 9.9 mmol.kgDM^−1^; p > 0.05 for all comparisons) (Fig. 3, Panel D).

### Prediction analysis

**Figure 4:**
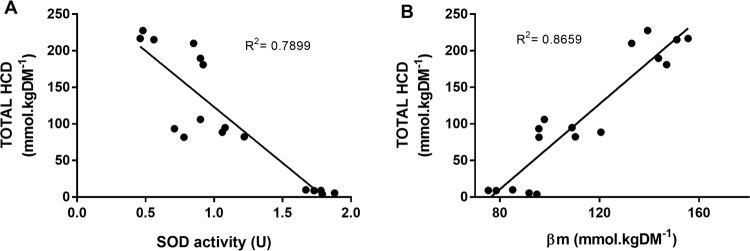
Coefficient of Determination Between Total HCD content and Superoxide Dismutase (SOD) Activity Panel (A), and Total HCD Content and βm Panel (B). Analyses are based on 17 samples, *i*.*e*., Hummingbird *m*. *pectoralis* n = 5; chicken *m*. *vastus lateralis* n = 6; chicken *m*. *pectoralis* n = 6.

Pearsons bivariate correlation analysis showed that total HCD content was strongly and significantly correlated with COX IV (R = −0.777; p < 0.001), SOD activity (R = −0.889; p < 0.001) and βm (R = 0.931; p < 0.001) but not with LDH (r = 0.327; p = 0.217). Multiple linear regression was used to identify the predictive value of these independent variables on the primary outcome (total HCD content). Only those variables that had a statistically significant bivariate correlation with total HCD were included in the model (namely COX IV, SOD activity and β_m_). Variables were entered using the stepwise method. COX IV did not statistically contribute to the model and was excluded. Both βm and SOD activity significantly contributed to the prediction of total HCD content (F(2, 14) = 118.3, p < 0.001, R^2^ = 0.944). The linearity of the correlation between βm and total HCD (Fig. 4, Panel B) confirms that βm_non-HCD_ is essentially the same in all three muscles, which, from the intersect with the y-axis, is approximately 80 mmol.kgDM^−1^.

## Discussion

The renowned physiologist August Krogh famously stated “For a large number of problems, there will be an animal of choice, or a few such animals on which it can be most conveniently studied”^24,25^. In recognition of the difficulty of studying isolated mechanistic pathways in *in-vivo* models, we employed a comparative physiology approach to examine the primary HCD role in skeletal muscle metabolism. The very low HCD content in the hummingbird tissue, which has remarkable oxidative and contractile properties, indicates that HCDs are not essential to these processes, and that their primary physiological function is unlikely to involve the primary reduction of reactive species, nor the regulation of Ca^2+^ transients or sensitivity. In contrast, HCDs were abundant in chicken *m*. *pectoralis* and *m. vastus* lateralis. These tissues (particularly the chicken *m*. *pectoralis*) have limited oxidative capacity and are, therefore, largely reliant upon anaerobic forms of energy metabolism that subsequently challenges pH homeostasis. Collectively, these findings indicate that the primary physiological function of HCDs, within skeletal muscle metabolism, is to act as intracellular physicochemical buffers.

Our assertion that the primary physiological role of the HCDs is to buffer H^+^ across the physiological pH range, is supported by the strong and positive linear relationship reported between total HCD content and βm (R^2^ = 0.8659; p < 0.001). Indeed, calculation of the HCD contribution to βm using the Henderson Hasselbalch equation^26^ showed that the variation in βm reported between the three muscle samples was due to variation in HCD content, and that βm_(nonHCD)_ was comparable between them (79.2 ± 9.9 mmol.kgDM^−1^). The HCD contribution to buffering capacity occurs as a result of the pKa of their imidazole rings (6.83 and 7.04 for carnosine and anserine), rendering them ideal buffers to maintain pH homeostasis within the intracellular environment, given that pH reduces from ~7.1 to ~6.5 during exhaustive exercise^27^, due to hydrogen cation accumulation^28^. A role in intramuscular proton buffering likely represents the mechanism underpinning previous findings reported in human muscle, *e.g.,* the preferential localisation of carnosine in Type 2 over Type 1 muscle fibres^29–31^, which are known to have a higher capacity for anaerobic metabolism, and therefore a higher buffering requirement. Additionally, supplementation with β-alanine (the rate limiting amino acid in HCD synthesis^32^) and the concomitant increase in intramuscular carnosine content enhances high-intensity exercise performance, mainly influencing capacity based assessments lasting between 3 seconds and 10 minutes^1^. These activities are strongly reliant on anaerobic metabolism and are therefore limited, at least in part, by a decrease in intramuscular pH. In contrast to the hummingbird *m*. *pectoralis*, the limited aerobic capacity of the predominantly glycolytic chicken *m*. *pectoralis* leads to an environment whereby acid-base regulation is regularly challenged, thus creating an evolutionary requirement for the development of an advanced system to protect pH homeostasis, which would explain the HCD abundance reported in this species. High carnosine content has also been reported in other species that have adapted to high-speed running as a means of either escaping predators (*e.g.,* horses), or capturing prey (*e.g.,* greyhound dogs)^26^. Survival in these species warrants an advanced ability to protect against the effects of high-intensity exercise induced acidosis, thus delaying fatigue and enhancing their ability to catch prey or escape predators. Similarly, the highest HCD content reported occurs in whales (~350 mmol^.^kgDM^−1^ ^33^) a mammal that undergoes prolonged hypoxia while diving, and thus relies heavily upon anaerobic metabolism. Collectively, the results of the current investigation, along with supporting evidence from both human and animal models strongly indicates that intracellular buffering is the primary biological role of the HCDs.

A striking finding of the current study was the strong and inverse relationship between HCD content and SOD activity (R^2^ = 0.7899), which appears to oppose the anti-oxidant role often attributed to carnosine^34^. Mitochondria are the primary source of superoxide radicals^35^, which are produced at a number of points along the electron transport chain^36^. Hummingbirds had significantly higher COX IV content compared to chickens (p < 0.001, Fig. 2, Panel A), which aligns with their higher mitochondrial content (~35% of cellular volume^37^) compared to chickens (~4%^38^) As a result, superoxide production would be high, necessitating a strong primary anti-oxidant system to prevent an accumulation of superoxide radicals (and other reactive species) and subsequent oxidative stress. Our results support this hypothesis, given that the SOD activity of the hummingbird skeletal muscle was substantially higher than either of the two chicken samples. Given that mitochondria are the primary source of reactive species^35^, while hummingbirds are known to have a very high mitochondrial content^37^, these results indicate that HCDs are unlikely to function as primary anti-oxidants, given the very low levels detected within the highly oxidative hummingbird tissue. This concept is supported by the literature, as *in vitro* experiments have previously shown that, while HCDs do have some primary anti-oxidant properties^39,40^, the effectiveness of this action is limited^6,40^. Interestingly, HCDs appear to be more effective at binding and removing secondary products of the anti-oxidant propagation cycle, *e.g.,* saturated and unsaturated aldehydes^41^. Indeed, emerging evidence indicates that the highly reactive HCD amine group, renders them capable of forming stable conjugates with lipid peroxidation products, including 4-hydroxynonel (4-HNE) and acrolein^42,43^. These products are only produced, however, when the primary anti-oxidant system is incapable of reducing primary oxidised particles, thus leading to a state of oxidative stress^35^. The strong primary anti-oxidant system of the hummingbird is likely to prevent the development of these secondary products, and therefore, the ability of the HCDs to bind and remove these products may be largely unnecessary in this species. Conversely, the limited aerobic capacity of the chicken *m*. *pectoralis* is likely to render them more susceptible to oxidative stress due to an inadequate primary anti-oxidant system, as demonstrated by the limited SOD activity in the muscles investigated within the current study. It seems plausible to suggest that protecting against the metabolic by-products of anaerobic metabolism, which may include the ability to form adducts with products of lipid peroxidation, such as 4-HNE and acrolein, may represent an important secondary role of HCDs. If so, this has important implications in conditions characterised by elevated oxidative stress and the accumulation of secondary propagation and lipid peroxidation products, including, for example, senescence, alzheimers disease and various cardiovascular conditions^41^.

*In vitro* evidence indicates that carnosine has the capacity to influence muscle contractility by enhancing the sensitivity of the sarcoplasmic reticulum Ca^2+^ release channels^44,45^. This is often proposed as a potential mechanism that may underpin the effect of carnosine on high-intensity exercise performance^7^, which is characterised by high contractile activity. Hummingbirds have the highest wingbeat frequency recorded for any avian species^11,12^, along with remarkable dexterity. Indeed, it has been proposed that the *m*. *pectoralis* of the hummingbird has two primary functions, namely locomotion and thermogenesis, both of which rely on calcium release and re-uptake from the sarcoplasmic reticulum Ca^2+^ pumps^46^. Given the low HCD levels in the hummingbird flight musculature, accompanied by their high reliance on Ca^2+^ release for locomotion and thermogenesis, it is unlikely that HCDs are primarily involved in the development of a highly functional muscle. Increased intramuscular acidity has been reported to influence sarcoplasmic reticulum Ca^2+^ release^47^ and, therefore, a role for HCDs in maintaining the pH of the intracellular environment may indirectly act to regulate muscle contractility during intense exercise.

The ratio of carnosine: anserine in these birds shown herein is interesting. Anserine is a carnosine analogue; the primary difference between these two molecules being that anserine has 1-methyl histidine in place of L-histidine, which is found in carnosine. The physiological relevance of the methylated versus the non-methylated forms of these HCDs is not clear. Anserine was consistently higher than carnosine in all of the muscle samples investigated in the current study (see Fig. 2). Conversely, carnosine is believed to be the only HCD in human skeletal muscle (~20–30 mmol^.^kgDM^−1^), although anserine is expressed in other human tissues (*e.g.*, the kidney^48^). Some functional differences between carnosine and anserine have been reported, including distinct pKa’s^49^ and protective properties against reactive species^50^. The distinct physiological role of these dipeptides, and the unique contribution of anserine versus carnosine to skeletal muscle metabolism, along with tissue-specific HCD effects, represent exciting research opportunities that warrant further investigation.

## Conclusion

In conclusion, our findings indicate that HCDs are non-essential to the development of a highly oxidative and contractile muscle, given their very low content in hummingbird *m*. *pectoralis*, indicating that their main physiological role is unlikely to involve protection against primary reactive species or the regulation of Ca^2+^. In contrast, HCD content was highest in chicken *m*. *pectoralis*, a tissue that has adapted to primarily metabolise substrates via anaerobic bioenergetic pathways, experiencing regular challenges to pH homeostasis as a result. Collectively, our results indicate that the primary mechanistic role of HCDs in skeletal muscle metabolism is to enhance anaerobic bioenergetics by acting as an intracellular physicochemical buffer.

## Methods

### Sample collection

Samples were collected from wild hummingbirds (*amazilia fimbriata*, n = 5) and free-living domestic chickens (*gallus domesticus*, n = 6). Wild-type and free-living birds were selected to replicate natural living conditions and behaviours as closely as possible. All hummingbirds were captured in the state of Sao Paulo Brazil, using mist nets (Ecotone® M-14/2). Permission to capture wild hummingbirds was obtained from the Brazilian Institute of Environment and Renewable Natural Resources (IBAMA no: 49347-2). Experimental procedures for this study were approved by the Bioscience Institute of the University of São Paulo Bioethics Committee (CEUA protocol number 222/2015), and all experiments were performed in accordance with all institutional and governmental guidelines and regulations. Hummingbirds were sacrificed using anaesthesia on the day of capture. Chicken samples were obtained from birds raised on a farm in the state of Sao Paulo. All chickens were sacrificed on-site by cervical dislocation. Following muscle dissection, all samples were flash frozen in liquid nitrogen, then stored at −80 °C until further analysis.

### Analytical procedures

#### HCD content

Total HCD content was determined using high performance liquid chromatography (HPLC) with UV detection (Hitachi Ltd., Tokyo, Japan), according to previously described methods^51^. Deproteinised muscle extracts were obtained from 3–5 mg of lypholysed samples^32^. All chromatography was carried out at room temperature. Deproteinized extracts (20 µL) were injected via an auto sampler using a cut injection method, and chromatographic separation was achieved using an Atlantis HILIC silica column (4.6 × 150 mm, 3 μm; Waters, Massachusetts, USA) attached to an Atlantis Silica column guard (4.6 × 20 mm, 3 μm). The method used two mobile phases: Mobile phase A (0.65 mM ammonium acetate, in water/acetonitrile (25:75) (v/v)), and mobile phase B (4.55 mM ammonium acetate, in water/acetonitrile (70:30)), with both solutions adjusted to pH 5.5 and filtered using a 0.2 μm filter membrane. The separation condition comprised a linear gradient from 0 to 100% of solvent B for 13 min at a flow rate of 1.4 mL∙min^−1^. Separation was monitored using an ultraviolet detector at a wavelength of 214 nm. Standard curves for carnosine and anserine were performed prior to analysis using concentrations of 0.1, 0.25, 0.5, 1, 2.5, and 5 mM, and regression equations generated using the area under the curve (AUC) of generated peaks (R^2^ = 0.999 and >0.999 for carnosine and anserine). Carnosine content was quantified by plotting the AUC of each sample against standard curve data, and reported as mmol·kgDM^−1^.

#### Aerobic and anaerobic enzyme content

The abundance of COX IV (a subunit of cytochrome C oxidase, and the terminal enzyme in the respiratory chain) and LDH (which catalyzes the inter-conversion of pyruvate and NADH to lactate and NAD^+^) were analysed using the Western Blot technique. Lysis buffer (RIPA, Sigma-Aldrich Ltd), with added phenylmethylsulfonyl fluoride (PMSF) (1 mM) and protease and phosphatase inhibitors were added to the muscle samples (10 µL of buffer·mg^−1^ of wet muscle). The muscle was homogenized using a bench-top motor driven homogenizer, with intermittent vortexing. The homogenate was then centrifuged at 12,902 relative centrifugal force (RCF), and the supernatant extracted. Total protein content was assessed using the Bradford technique, and the results used to calculate the quantity of Laemli loading buffer required (1:5 µl:µg). Samples were separated by SDS-PAGE in pre-cast polyacrylamide gels (4–20%, Bio Rad Laboratories Inc), with equal loading in each well (30 µg of protein). A molecular mass marker (Precision Plus TM Dual Color Standards, Bio Rad Laboratories Inc) was used to locate the proteins of interest (COX IV: 17 kDa; LDH: 37 kDa). Proteins were transferred to a nitrocellulose membrane using gel electrophoresis (75 minutes at 100 V, 4 °C). Total protein transfer was visualised using Ponceau staining (Supplementary File 1), and the membranes were then washed for 3 × 10 minutes in TBS-T, followed by a 10 min wash in TBS. The membranes were blocked in a 5% blocking solution (TBS-T with non-fat milk powder) for 2 hours then the wash procedure was repeated. Membranes were then incubated with the primary antibody for 12–14 hours at 4 °C (Cell Signalling Technology® #2012 (LDHA) & 4850 (COX IV; 3E11)). Following the primary antibody incubation, membranes were washed as described above, then incubated in an anti-rabbit, horseradish peroxidise (HRP) linked secondary antibody (Cell Signalling Technology® # 7074 S). Excess secondary antibody was washed, and the membranes were exposed to an enhancing solution at room temperature, then visualised by chemiluminescence using the Image Quant LAS 4000 (GE Healthcare®). Protein content was quantified using densitometry (Scion Image®), and all bands were normalised to a positive control comprising human *m*. *vastus lateralis*, with 40 mg of protein.

#### Superoxide dismutase (SOD) activity

Superoxide dismutase (SOD) activity was measured based on the inhibition of xanthine/xanthine-oxidase-driven cytochrome C reduction by the activity level of SOD^52,53^. Approximately 25–35 mg of wet muscle was homogenized in phosphate buffer (50 mM KH_2_PO_4_, 50 mM K_2_HPO_4_, pH 7.8, protease inhibitors; 1:4 mg:µL), using a bench-top motor driven homogenizer. The homogenate was centrifuged at 12,902 RCF for 15 min, and the supernatant used to measure SOD activity. Protein content was measured using the Bradford technique. Initially, the rate of cytochrome C reduction was measured in the absence of sample for 5 minutes at an absorbance of 550 nm, through mixing a solution containing EDTA (0.1 mM), xanthine (50 µM) and cytochrome C (20 µM) and xanthine oxidase (diluted in the phosphate buffer (50 mM), with another solution containing phosphate buffer (50 mM) with added EDTA (0.1 mM) and xanthine oxidase (0.2 U·ml^−1^). Subsequently, the cytochrome C reduction rate was measured in the presence of the sample homogenate, containing 20 µg of protein. SOD activity was calculated according to the difference in the two reduction rates, and measured within the linear range of the assay. SOD activity is expressed in units (U), whereby 50% inhibition of the cytochrome C reduction rate was considered to represent 1U of SOD activity.

#### Muscle buffering capacity (βm

The non-bicarbonate skeletal muscle buffering capacity (βm) was assessed using the homogenate titration technique, as previously described^54^. Whole muscle samples were lyophilised, then extracts of powdered tissue (approximately 2.5–3.5 mg) were homogenized by intermittent vortexing (5 × 20 s passes, interspersed with 30 s periods on ice) in a non-buffered NaF solution (10 mM; 30 mgDM·ml^−1^ ^55–60^). Samples were subsequently equilibrated for 5 minutes at 37 °C and with constant motion, using a thermomixer. The pH was measured using a micro-electrode (InLab Micro; Mettler Toledo) connected to a pH meter. Prior to starting the experiment, the pH of the muscle homogenates was adjusted to 7.1, using an NaOH solution (0.02 M). Homogenates were subsequently titrated to pH 6.5 through the repeated addition of 2 µL of a HCI solution (10 mM). The total amount (moles) required to change the pH from 7.1 to 6.5 was recorded, and the value normalised to the starting weight (kg) of dry tissue used in each experiment (mmol.kgDM^−1^). The HCD contribution to total muscle buffering capacity was calculated using a derivation of the Henderson-Hasselbalch equation, as previously described^26^ according to the calculation:

$${{\rm{\beta }}}_{{\rm{HCD}}}=\{[{\rm{HCD}}]/(1+{10}^{(6.5-{\rm{pKa}})})\}-\{[{\rm{HCD}}]/(1+{10}^{(7.1-{\rm{pKa}})})\}$$

where [HCD] is the content of either carnosine and anserine in mmol.kgDM^−1^, and assuming a pKa of 6.83 and 7.04 for carnosine and anserine^49^.

For each sample, the buffering contributed specifically by carnosine and anserine between pH 7.1 and 6.5 was calculated using the above equation, and the sum of the HCD contribution to βm was subtracted from the total muscle buffering capacity to provide a measure of the non-HCD buffering capacity, *i*.*e*. β_non-HCD_ = β_m_ − β_HCD_.

### Statistical Analysis

Data were analysed using the Statistical Package for Social Sciences (SPSS version 17.0). One way ANOVA, with tukey post hoc adjustment, was used to locate differences between the three muscle types (hummingbird *m*. *pectoralis*, chicken *m*. *vastus lateralis*, chicken *m*. *pectoralis*) for all outcome measures. Bivariate correlation analysis between all independent variables (COX IV, LDH, SOD activity and βm) and the dependent variable (total HCD content) was conducted using Pearson’s correlation coefficient. Multiple linear regression was used to identify the predictive contribution of these independent variables on the primary outcome of interest, namely total HCD content. Variables were entered using the stepwise function, which functions by building a predictive model through adding or removing variables based on the t-statistics of their estimated coefficients. Statistical significance was accepted at the level of p < 0.05. All outcomes are reported as mean ± 1 SD.

## Electronic supplementary material

Supplementary File 1: Western Blot Images

## Data Availability

The datasets generated during and/or analysed during the current study are available from the corresponding author on reasonable request.
