# Supplementary material for: A Comparative Study of Hummingbirds and Chickens Provides Mechanistic Insight on the Histidine Containing Dipeptide Role in Skeletal Muscle Metabolism
Source: Sci Rep. 2018 Oct 3;8:14788. doi: 10.1038/s41598-018-32636-3 (PMC6170442; doi:10.1038/s41598-018-32636-3)
Supplement: Supplementary file 1 — Western Blot Images [file 41598_2018_32636_MOESM1_ESM.pdf]

SUPPLEMENTARY FILE ONE: Western Blots Images

A Comparative Study of Hummingbirds and Chickens Provides Mechanistic Insight on the Histidine Containing Dipeptide Role in Skeletal Muscle Metabolism

*Dolan E[1], Saunders B[1], Dantas WS[1], Murai IH[1], Roschel H[1], Artioli GG[1], Harris R[2], Bicudo JEPW[3], Sale C[4], *Gualano B[1].*

1: Applied Physiology and Nutrition Research Group, Rheumatology Division; Faculdade de Medicina FMUSP, Universidade de Sao Paulo, Sao Paulo, SP, BR, University of São Paulo, SP, BR.
2: Junipa Ltd; Newmarket; Suffolk; United Kingdom.

3: School of Biological Sciences; University of Wollongong; Australia.

4: Sport, Health and Performance Enhancement Research Centre; Musculoskeletal Physiology Research Group; School of Science and Technology; Nottingham Trent University; United Kingdom.

Corresponding Author:

Professor Bruno Gualano

Applied Physiology & Nutrition Research Group.

Rheumatology Division,

Faculdade de Medicina FMUSP, Universidade de São Paulo,

Sao Paulo, Brazil.

Phone: +55 11 2648-1337; Fax: +55 11 3061-7490;

E-mail: gualano@usp.br

Complete COX IV (17kDa) gels:

Gel 1:

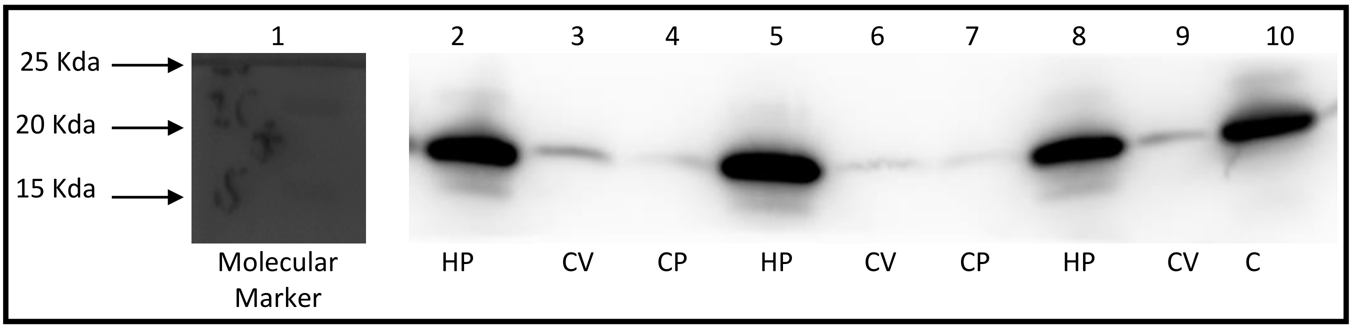

Gel 2:

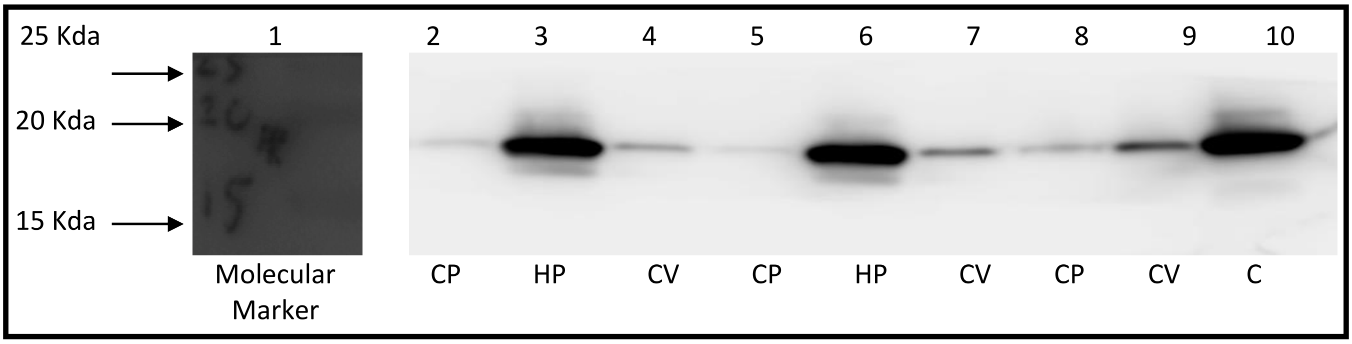

HP: Hummingbird *m. pectoralis*; CV: Chicken *m. vastus lateralis*; CP: chicken *m. pectoralis*; C: Control (human *m. vastus lateralis* with 40mg of protein)

Complete LDH (37kDa) gels:

Gel 1:

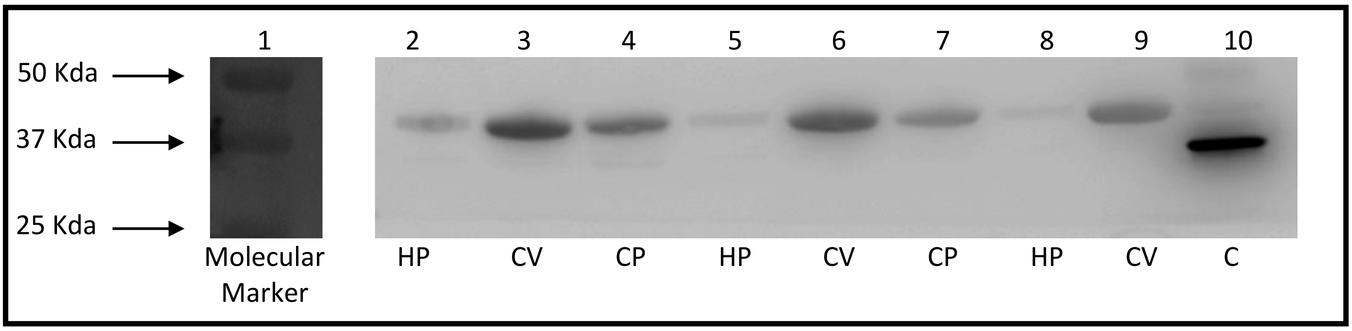

Gel 2:

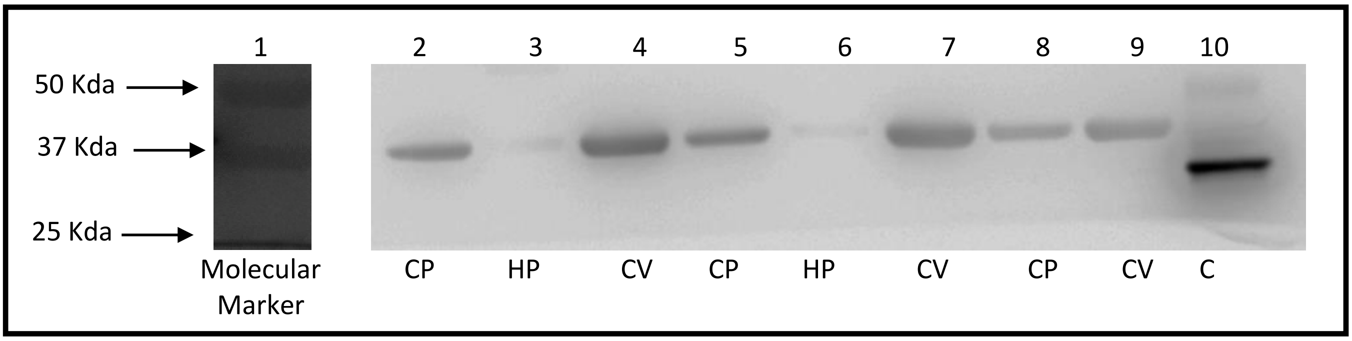

HP: Hummingbird *m. pectoralis*; CV: Chicken *m. vastus lateralis*; CP: chicken *m. pectoralis*; C: Control (human *m. vastus lateralis* with 40mg of protein)

Protein Transfer visualised using Ponceau Staining

Gel 1: **Gel 2:**

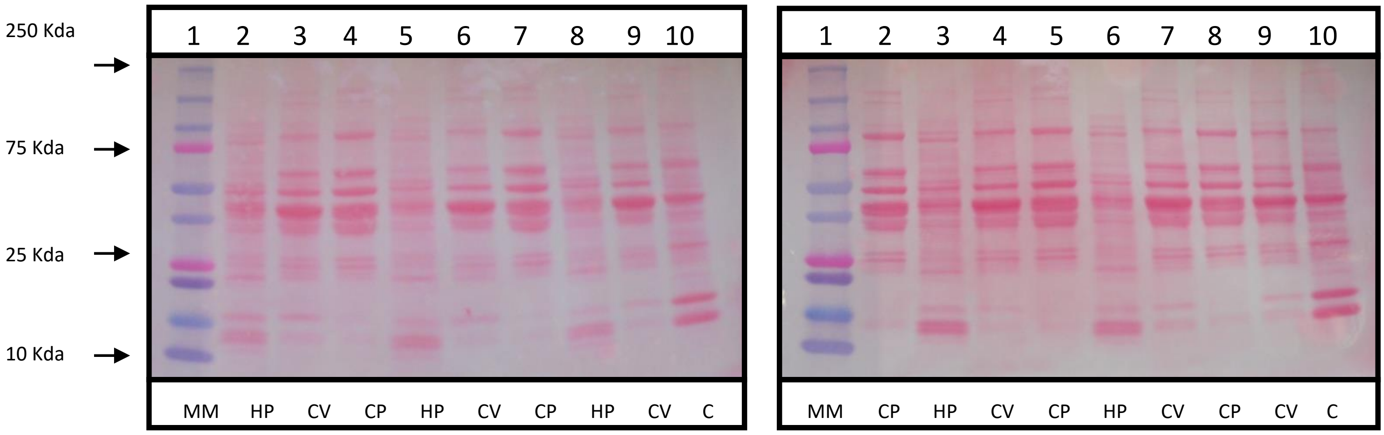

MM: Molecular Mass Marker; HP: Hummingbird *m. pectoralis*; CV: Chicken *m. vastus lateralis*; CP: *Chicken m. pectoralis*; C: Control (human *m. vastus lateralis* with 40mg of protein)
